# A novel approach to surviving an acute aorto-oesophageal fistula: A case report

**DOI:** 10.1016/j.ijscr.2024.110035

**Published:** 2024-07-14

**Authors:** Kyle R. Joseph, Jyotindra Singh, Ronald Chin, Arvind Lee, Yana Oborska, Yaroslav Mayorchak

**Affiliations:** a Nepean Hospital, Australia; b Nepean Private Hospital, Australia

**Keywords:** Aorto-oesophageal fistula, Haematemesis, Foley catheter, Endovascular surgery, Thoracic surgery

## Abstract

**Introduction and importance:**

Acute aorto-oesophageal fistula poses a significant mortality risk, requiring immediate and decisive medical intervention. This report highlights the critical need for innovation in emergency surgical responses.

**Case presentation:**

A 57-year-old male, with a history of aortic repair, presented with chronic anaemia and dysphagia. He suffered a cardiac arrest from massive hematemesis during surgery for an infected thoracic hematoma. Lacking a Stengsten-Blackmore tube, a 26Fr Foley catheter was used to control the bleeding. This measure stabilized the patient enough for a definitive endovascular repair with aortic stents, which successfully managed the bleeding.

**Clinical discussion:**

The treatment objectives for this condition include initial control of oesophageal bleeding, followed by endovascular management to further control the bleeding, subsequently releasing the oesophageal control, and ultimately preventing infection through the administration of intravenous antibiotics.

**Conclusion:**

This case illustrates the importance of adaptability and the use of unconventional methods in emergency situations, demonstrating that innovative solutions can be lifesaving in critical surgical emergencies.

## Highlights

• Aorto-oesophageal Fistula is immediately life-threatening.
• A Foley Catheter (with 30ml of saline in balloon) can be used to tamponade bleeding in the oesophagus.
• Tamponade transiently maintains stability until the patient can have definitive endovascular treatment.

## 1 Introduction

The list of pathologies more dramatic and more deadly than acute haematemesis due to an aorto-oesophageal fistula (AOF) is very, very short [1,2]. Trained specialists in theatre or in an emergency department can be forgiven to be taken aback by continuous projectile vomit of frank blood. The mortality for such a condition is inevitable if bleeding is not controlled in an effective and timely manner [3,4]. The main causes for AOF include thoracic aortic aneurysm (54 %) and ingestion of corrosive foreign material (such as button batteries) seen in 19 % of cases [5,6], there have also been some cases caused by ingestion of fish/animal bones [3,7,8]. Malignancy is another cause. Mortality, if treated conservatively, approaches 100 % with mortality post-surgical intervention also being as high as 40 % [9]. The classic triad of mid-thoracic chest pain, sentinel arterial haemorrhage, and fatal haemorrhage has become known as aorto-oesophageal syndrome, and can range from 45 to 80 % of patients [10].

Due to its rarity, there have been only a few case reports without any major randomised control trials. Current treatment approaches rely on clinicians having the time to arrange operative intervention before the patient exsanguinates completely. The first case of survival was reported 1983 by Snyder and Crawford [11]. Current management relies on early intervention and conservative treatments have no late survival [12]. The current case report details the life-saving technique of using a Foley's catheter to tamponade the bleeding while endovascular repair could be arranged. It has been reported in line with the SCARE criteria for surgical case reports [13].

## 2 Case report

### 2.1 Preoperative function including past medical history

KW, a 57-year-old male, had a history of a descending aortic false aneurysm following a motor vehicle accident (MVA) 27 years ago. A persistent leak into the aneurysm resulted in multiple surgeries over many years to stent and decompresses the collection. He underwent an open descending aorta replacement under circulatory arrest, which was subsequently complicated by a left-sided empyema, which required drainage and thoracic decortication. He recovered well from this and went on 3 months of IV antibiotics.

### 2.2 Current presentation

**Fig. 1:**
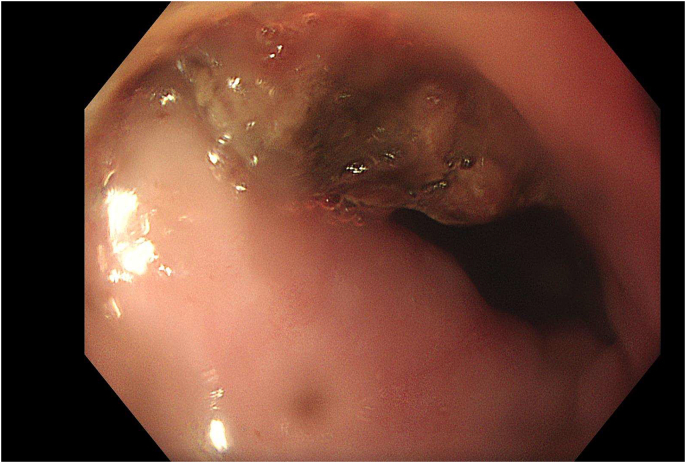
Endoscopic finding of fibrin clot in the oesophagus at 28 cm from the incisors.

Approximately 6 months after finishing his course of antibiotics, KW presented to hospital for investigation of chronic anaemia and dysphagia. Gastroscopy revealed a fibrin clot within the anterior wall of the oesophagus at ~28 cm from the incisors (Fig. 1). Repeat contrast CT did not reveal an obvious fistula but showed signs of gas and an increasing size of the collection, which raised concerns of a potential infection. The patient was treated with cefepime, yet his symptoms of dysphagia, and chronic anaemia persisted.

Repeat imaging, again, did not suggest a fistula. The case was discussed within a multidisciplinary team consisting of cardiothoracic surgeons, upper GI surgeons and vascular surgeons. The consensus was that surgical drainage was necessary to evacuate any remaining hematoma, address sepsis, and obtain cultures for sensitivities.

The drainage of the collection, which was found to be an old hematoma, was carried out via a left-sided single-port (2.5 cm incision) VATS technique. During the procedure, minor bleeding was encountered but promptly controlled with sutures. However, upon closure, the patient's blood pressure was found to be extremely labile. The anaesthetist observed blood being expelled from the patient's mouth at an alarming rate, and the patient experienced cardiac arrest on the operating table, with ~5 min downtime. After positioning the patient supine, cardiopulmonary resuscitation and a massive transfusion protocol was initiated, involving O-negative blood, fresh frozen plasma, and cryoprecipitate. A number of surgeons were present including Upper GI, General, ENT and Cardiothoracic surgeons.

A Stengstaken-Blackmore tube was not available at the time. Bleeding was controlled with per oral insertion of a 26Fr Foley catheter and inflation of the balloon with 30 ml of saline, successfully tamponading the bleeding.

**Fig. 2:**
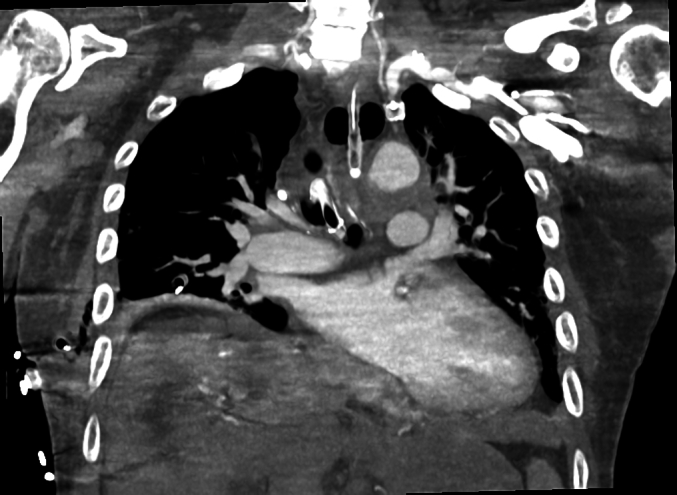
Coronal section of CT chest performed with IDC in situ, occluding the fistula.

**Fig. 3:**
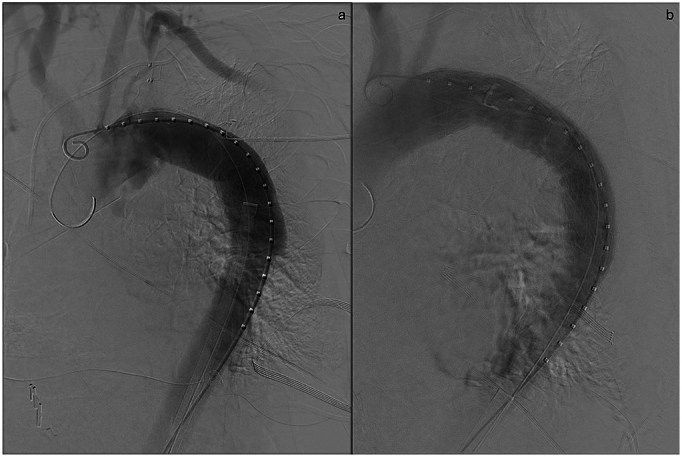
Fluoroscopic imaging of arch of the aorta before (a) and after (b) stenting.

The patient was then transferred to the ICU, where he was re-imaged (Contrast CT revealed no leak/fistula with the Foley catheter in situ; Fig. 2) and arranged for a vascular surgeon to perform an endovascular stent graft repair of the aorta. This was performed using both a Gore TAG 31x31x150 stent and Gore 34x34x150 stent with overlap in the thoracic aorta. A Coda balloon (32 mm) was used to get stent wall apposition and cease haemorrhage. The Foley catheter balloon was then deflated with no further exsanguination (Fig. 3). Post operative Gastroscopy confirmed the cessation of the leak prior to discharge. The patient was successfully discharged from hospital on light activities within a week. The patient has followed up in 4 weeks with the cardiothoracic surgeon with a repeat CT Chest showing no leak from the fistula.

## 3 Discussion

AOF is rare, accounting for only 4 % of all aorto-enteric fistulae [14]. Primary AOF results from communication of the oesophagus with an aortic aneurysm, whereas secondary AOF develops post stent/graft insertion into the aorta [15]. The incidence of primary AOF has remain relatively constant across time (0.04–0.07 %), whereas the incidence of Secondary AOF is seen to be increasing with the number of endovascular aortic surgeries that are performed (0.6–2.3 %) [1,14,15].

There are a number of theories as to why such an AOF forms, this includes oesophageal ischemia caused by pressure on the oesophageal artery, with simultaneous pressure on an aneurysm on the posterior mediastinum, or alternatively inflammation during haematoma resorption and anatomical changes to the aortic arch and descending aorta following placement of endovascular stents or grafts [[15], [16], [17], [18]]. Another mechanism results from perioperative infection of an endovascular prosthesis that results in pseudoaneurysm formation and pressure being exerted on the oesophagus resulting in the formation of the AOF [1,2,19]. In KW's case it seems to be a combination of both mechanisms.

The aims of treatment involve firstly control of bleeding via the oesophagus, control of bleeding via an endovascular approach, release control on the oesophagus and finally prevention of infection with intravenous antibiotics. Management of oesophageal bleeding has been typically with the use of Stengstaken-Blackmore Tubes to tamponade the bleeding until endovascular repair can be achieved [20]. Endovascular repair is typically performed with a stent graft placed into the aorta to cover the opening of the fistula.

Open repair of the aorta may also be definitive in some cases [21,22]. Surgical access is usually achieved with a left lateral thoracotomy, or by midline sternotomy in rare cases [9]. The aim was to gain access to the fistula and aortic arch to perform an extra-anatomic bypass on patients. Such access also allows for the placement of a pleural interposition flap to prevent recurrence of the fistula. If an operating room consists of a hybrid theatre, an immediate conversion from endoscopic to open would be beneficial. Mortality from surgical intervention can range vary from 20 to 93 % [14,23,24]. Gastroscopic assessment of the bleeding can range in sensitivity from 25 to 80 % [23]. If a clot is found on the oesophageal wall, the recommendation is to not remove or disrupt the clot.

In KW's case, despite the incident occurring intraoperatively, there was greater concern of exacerbating the hole in the aorta, and causing fatal bleeding and thus the Foley catheter was inserted to the oesophagus to tamponade the bleeding.

## 4 Conclusion

The successful outcome, with KW's discharge from the hospital within a week of the event, reflects the value of swift and coordinated efforts in a high-stakes surgical scenario. This case also underscores the significance lateral thinking and improvisation, especially when equipment is unavailable or open operation is too dangerous. We have demonstrated conclusively that a Foley Catheter can be used to tamponade life-threatening bleeding from an aorto-oesophageal fistula.

## Consent

The patient has signed a valid consent to allow their details to be used in this case report.

## Ethical approval

Approval is Except for the Current Case in our institution (Nepean Hospital; Thoracics Department). This case was performed as an emergency case as the patient was deteriorating rapidly. Consent was attained from the patient at the time of surgery and post recovery for the case report.

## Funding

The authors did not receive any funding for the current case report.

## Author contribution

Kyle R Joseph – Writing the Paper

Ronald Chin – Management Design

Jyotindra Singh – Data Collection

Arvind Lee – Management Design

Yana Oborska – Data Interpretation

Yaroslav Mayorchak – Editing, Study concept and design

## Guarantor

Yaroslav Mayorchak.

## Declaration of competing interest

We declare there are no competing or conflicts of interests in relation to the current case report.
